# β3-Adrenoceptors as Putative Regulator of Immune Tolerance in Cancer and Pregnancy

**DOI:** 10.3389/fimmu.2020.02098

**Published:** 2020-09-02

**Authors:** Maura Calvani, Annalisa Dabraio, Angela Subbiani, Daniela Buonvicino, Veronica De Gregorio, Sara Ciullini Mannurita, Alessandro Pini, Patrizia Nardini, Claudio Favre, Luca Filippi

**Affiliations:** ^1^Department of Paediatric Haematology-Oncology, A. Meyer University Children’s Hospital, Florence, Italy; ^2^Department of Health Sciences, University of Florence, Florence, Italy; ^3^Department of Experimental and Clinical Medicine, University of Florence, Florence, Italy; ^4^Neonatal Intensive Care Unit, Medical Surgical Feto-Neonatal Department, A. Meyer University Children’s Hospital, Florence, Italy

**Keywords:** beta-blockers, beta-adrenergic, fetal immune tolerance, cancer immune-tolerance, embryo implantation

## Abstract

Understanding the mechanisms of immune tolerance is currently one of the most important challenges of scientific research. Pregnancy affects the immune system balance, leading the host to tolerate embryo alloantigens. Previous reports demonstrated that β-adrenergic receptor (β-AR) signaling promotes immune tolerance by modulation of NK and Treg, mainly through the activation of β2-ARs, but recently we have demonstrated that also β3-ARs induce an immune-tolerant phenotype in mice bearing melanoma. In this report, we demonstrate that β3-ARs support host immune tolerance in the maternal microenvironment by modulating the same immune cells populations as recently demonstrated in cancer. Considering that β3-ARs are modulated by oxygen levels, we hypothesize that hypoxia, through the upregulation of β3-AR, promotes the biological shift toward a tolerant immunophenotype and that this is the same trick that embryo and cancer use to create an aura of immune-tolerance in a competent immune environment. This study confirms the analogies between fetal development and tumor progression and suggests that the expression of β3-ARs represents one of the strategies to induce fetal and tumor immune tolerance.

## Introduction

### Starting From Immune Tolerance

Immunological privileges such as immune tolerance represent the most powerful mechanism that preserves life. Understanding the mechanism of immune tolerance can lead to new strategic therapies in several contexts, as in minimizing the use of toxic drugs in transplants and in establishing more effective immune responses and vaccines for cancers and infection.

Cancer and embryo share similar mechanisms to sustain their progression: both (i) grow in a hypoxic and catecholamine-rich environment and (ii) tolerate a “foreign body” by creating an immune-tolerant microenvironment.

### Immune Tolerance in Fetus and Cancer

The maternal immune tolerance is one of the most intriguing and powerful mechanisms in current biology. During pregnancy, the maternal immune system actively tolerates embryo alloantigens, leading to fetus development (1). At the beginning of pregnancy, after conception, the endometrium converts into decidua to promote embryo implantation and the interface between fetus and maternal tissues becomes an immunologically privileged site (2). Several immune cells in the subpopulation recruited at the fetal–maternal interface are involved in maternal immune tolerance.

Recent data show that successful pregnancy requires not only fetal but also placental immune tolerance, contributing to the promotion of an immune-tolerant environment for the fetus. The human fetus is continuously exposed to self-antigens, maternal alloantigens, and nutritional antigens transferred across the placenta that its immune system must learn to tolerate. Moreover, the human placenta, although not an immune organ by definition, is highly active in promoting an immune-tolerant environment. Several different immune subpopulations are currently under investigation for the study of immune tolerance in both pregnant women and cancer patients. Actually, cancer is a foreign body for the host and thus different immune subpopulations are needed to sustain an immune-tolerant microenvironment. Here, we proposed a similarity between placenta and tumor microenvironment (TME) in promoting immune tolerance.

Among the different subpopulations involved in fetal and cancer immune tolerance, myeloid-derived suppressor cells (MDSCs) are activated at the fetal–maternal interface by interaction with trophoblast cells, and suppress T cell activation promoting Foxp3 expansion. In cancer, MDSCs induced the upregulation of IL-10 that downregulates macrophage IL-6 and IL-12 and tumor necrosis factor (TNFα) production, thereby polarizing tumor-associated macrophages (TAMs) toward a tumor-promoting M2 phenotype (3–5). Furthermore, cancer MDSCs block natural killer (NK) activity and their INF-γ secretion leads to anergic NK (6). Recent studies have shown that MDSCs and TAMs can promote angiogenesis by the induction of matrix metallopeptidase 9 (MMP9), vascular endothelial growth factor (VEGF), and IL-1β (7–13).

Regulatory T cells (Treg) are the predominant cells in both pregnancy and cancer and confer immunologic protection to embryo and cancer. Immune-suppressive maternal Foxp3^+^ Treg cells, detected at the fetal–maternal interface are critical to create and maintain a fetal–maternal-tolerant microenvironment by blocking alloreactive Th1 cells (14, 15). An altered Th1/Th2 cytokine balance with Th2 predominance and T-cell transient anergy makes the placental microenvironment an immunologically privileged site (16). Moreover, Treg cells participate in indoleamine-2,3-dioxygenase (IDO) (17) and placental heme oxygenase (HO)-inducible isoform expression, engaged in Foxp3-mediated immune suppression (18).

Recently, it has been reported that Treg cells accumulated in the human and murine decidua constitutively express cytotoxic T-lymphocyte antigen 4 (CTLA-4) (19, 20) and inhibit the interactions between CD28 expressed on T cells and their ligands, B7-1 and B7-2, expressed on antigen-presenting cells, such as macrophages or dendritic cells. Blockade of this interaction has been shown to induce antigen-specific peripheral tolerance (21–23). Fetal-specific Treg cells persist also after delivery, maintain tolerance to preexisting fetal antigens, and rapidly re-accumulate during subsequent pregnancy. Therefore, pregnancy imprints a sort of regulatory memory through the specific maternal Treg cells (24). Interestingly, a high number of maternal cells cross the placenta and, in fetal lymphoid tissues, induce the development of Treg cells (25).

In the human fetus, Treg cells are precociously detected, as early as 13 weeks of gestation (26). Their prevalence is significantly higher in fetal lymphoid tissues (on average, 15–20% of CD4^+^ T cells) than that observed in adult lymph nodes (usually less than 5%), and these cells are able to suppress the proliferation and function of both CD4^+^ and CD8^+^ T cells (27). Moreover, Treg cells induce immune suppression through the production of inhibitory cytokines, such as Transforming Growth Factor beta (TGF-β), IL-10, and IL-35 (28, 29), depleting the availability of IL-2, or killing the effector or Antigen-Presenting Cells (APC), thanks to the upregulation of perforin, production of granzyme B, or interaction with Fas/FasL (Fas Ligand) (30).

Furthermore, Treg cells are activated by the ICOS (inducible T−cell co-stimulator)−ICOSL (ICOS ligand) and programmed cell death-1 (PD−1)/PD-ligand 1 (PD-L1) pathways in conjunction with the inhibition of effector T cells by the lymphocyte activation gene−3 (LAG−3)−MHC class II pathway (31). The interaction between CTLA−4 expressed by Treg cells and CD80/86 on APCs promotes IDO secretion (32). It is well known that the expression of IDO and tryptophan 2,3-dioxygenase (TDO) leads to tryptophan depletion in the TME and causes T cell dysfunction (33).

Natural killer cells represent the majority of immune cells present in the fetal–maternal interface of the pregnant uterus, where they show a specific function and a peculiar phenotype during pregnancy (34). While circulating conventional natural killer (cNK) cells are cytotoxic lymphoid cells programmed to have an active role in promoting leukocyte activation and immune surveillance against infections and cancer (35), distinct subsets of resident NK cells have been described in specific tissues, such as the uterus (36). In contrast to cNK cells, NK cells detected in the decidua during pregnancy, referred to as decidual NK (dNK), appear to be primarily responsible for promoting placentation (37), as suggested by the expression of specific inhibitory receptors (KIR) and poor cytotoxic activity (38).

Decidual NK show a distinct phenotype compared to peripheral blood. In fact, despite that dNK have abundant intracellular granules containing granzymes, granulysin, and perforin, they are poorly cytotoxic, probably as a consequence of the recognition of human leukocyte antigens-alpha chain E (HLA-E) expressed on trophoblasts (39), even though dNK cell cytotoxicity can increase in an inflammatory environment (40). dNK appear to be involved in the promotion of immune tolerance, thanks to the interaction with decidual myelomonocytic CD14(+) cells which induce Treg cell expansion, through the expression of IDO, the production of TGF-β, or an interaction mediated by CTLA-4 (41).

Moreover, NK infiltration represents instead a positive prognostic marker in cancer cells, due to their cytotoxic activity (42–44), but unfortunately, frequently the number of infiltrated NK is reduced, and their activity is not sufficient to counteract tumor progression (45, 46).

### β-Adrenergic System and Immune Regulation

Stress, catecholamine synthesis, and β-adrenergic receptors (β-ARs) have long been investigated as regulators of many physiological processes, including cardiac and pulmonary physiology and immune responses. The effects of catecholamine epinephrine and norepinephrine are mediated by β-ARs which belong to the G-protein-coupled receptors family and classified into three subtypes widely expressed in various tissues: β 1-, β2-, and β3-AR. It is well known that β-AR signaling is involved in the regulation of several cellular processes that contribute to cancer initiation and progression (47–50): in particular, downregulation of antitumor responses and accumulation of immunosuppressive cells, including TAMs and MDSCs, is induced by stressful conditions. Several *in vitro* and *in vivo* studies have demonstrated the behavioral stress and catecholamine involvement in promoting cancer progression through decreased NK activity and immune suppressive effects (51–57). Norepinephrine, the β-AR agonist isoproterenol, and the β2-AR selective-agonist metaproterenol inhibit NK cell cytotoxic activity in splenocytes, by downregulating perforin, granzyme B, and IFN-γ at the mRNA and protein levels (58). Similarly, stress due to immobilization in rats induces an upregulation of catecholamines and, consequently, a reduction in NK cytotoxicity (59). Moreover, Shakhar G. et al. have shown that β-AR agonism remarkably suppresses NK activity and this compromises host resistance to mammary adenocarcinoma MADB106, an NK-sensitive tumor, in rats (60). The same result is observed in CRNK-16 leukemia where stress leads to suppression of NK activity sufficient to promote tumor development (61). In human patients, apparent conflicting results of clinical studies have been reported: elevated NK activity was reported after epinephrine infusion (62), open-heart surgery (63), or physical exercise (64). However, subsequent studies suggested that this increase was attributable to a marked, but transitory, increase in the number of circulating NK cells, rather than to an increase in activity per NK cells (65). The increase in circulating number of NK cells occurs during the time of elevated catecholamine levels and dissipates shortly after their decline (66).

Recently, β2-AR has been detected on Treg cells. β2-AR signaling, following norepinephrine stimulation, improves the suppressive properties of Treg cells, associated with a decrease in IL-2 expression, and increases the expression of CTLA-4, a molecule that promotes T-cell anergy, improving Treg cell suppressive function in a PKA-dependent manner. In addition, β2-AR signaling stimulates Treg-cell-mediated conversion of CD4^+^ Foxp3^–^ cells (memory T-cells) into Foxp3^+^ iTreg (induced Treg) cells, in a PKA-dependent manner, improving Treg cells’ suppressive function (67). Moreover, MDSCs have been reported to be increased in mice exposed to chronic stress (68) and in patients who reported high levels of stress, suggesting that they may be a contributing factor to the immune suppression as observed in breast cancer patients (69). Experimental studies demonstrated that *in vitro* treatment with norepinephrine significantly enhanced the expansion of the MDSC population, resulting in suppression of T-cell proliferation, suggesting a role of catecholamines in myeloid cell differentiation and function (70).

In summary, the current literature suggests that β-adrenergic activation promotes immunosuppression, as indirectly confirmed by the increased survival rate and the improved response to immunotherapy in melanoma patients (71). However, so far, the focus has been almost exclusively on β2-AR. Recently, a great interest has accrued regarding the role played by the β3-AR in the promotion of fetal and cancer growth and in the induction of an immune-tolerant environment.

### β-Adrenergic System and Fetal and Cancer Development

The role of β-adrenergic signaling in pregnancy and the cancer microenvironment is widely reported (47, 72, 73).

Catecholamines are required for mouse fetal development and postnatal survival, as demonstrated by lethality at mid-gestation after blocking their biosynthetic pathway (74, 75). Moreover, during fetal development, catecholamines modulate fetal circulation in hypoxic conditions by reducing the fetal heart rate (72, 73) and preserve heart and brain glucose homeostasis, and their increase at birth is essential to neonatal adaptation, for example to facilitate delivery and induce surfactant production (72, 73, 76).

Several studies show that catecholamines released during stress and β-AR signaling are able to regulate multiple cellular processes that accelerate tumor progression, including cancer cell growth, migration, and angiogenesis, leading to reduction in patient overall survival (47, 51). Among β-ARs, β_2_-AR is considered the principal receptor subtype involved in the modulation of catecholamine effect in cancer (77), and it may explain why non-selective β-AR blockers (acting on β1- and β2-AR) provide protection against different types of cancer (78–80).

### β3-Adrenergic Receptor in Fetal and Cancer Development

The roles played by β3-AR in embryonic development and fetal life remain poorly understood. However, studies report β3-AR expression in human and animal germ cells, where it induces motility (81), in pre-implantation embryos (82, 83), during the first stages of embryogenesis (84), in embryo tissues, and in placenta (85, 86). Moreover, β3-AR is upregulated in the human pregnant myometrium where inhibits spontaneous contractions and represents the predominant subtype over β2-AR (87, 88). These data suggest a role of β3-ARs in the promotion of fecundation, embryo implantation, and growth.

Recently, a growing number of studies have demonstrated the emerging role of β3-AR signaling in cancer development and progression. β3-AR expression has been reported in different tumors, including colon cancer (89), leukemia cells (90), and human vascular tumors (91). In addition, the Trp64Arg polymorphism in *ADRB3* (β3-AR gene) was reported to be associated with susceptibility to endometrial cancer and decreased risk for breast cancer, especially when associated to Gln27Glu polymorphism in *ADRB2* (β2-AR gene) (92, 93). A recent study in β1-, β2-AR, and β1/β2-AR knockout mice has suggested that not only β2- but also β3-AR result to be actively involved in prostate cancer development (94). Moreover, in melanoma B16F10 cells, we have demonstrated that β3-AR is expressed and significantly upregulated after the exposure to hypoxia, promoting VEGF production in a nitric oxide (NO)-mediated manner. In mice bearing melanoma, we have recently reported that β3-AR blockade reduces tumor volume and the development of tumor vasculature, through decreased cell proliferation and increased apoptosis of melanoma cells (95–97). Recently, the correlation between β3-AR expression and melanoma aggressiveness has been demonstrated in human melanoma tissue samples. This study, for the first time, detected β-AR expression not only on the surface of cancer cells but also in stromal, inflammatory, and vascular cells of TME, where β3-AR was able to enhance melanoma cells, to respond to environmental stimuli, to increase cancer cell motility, and to induce stem-like traits. Finally, β3-AR stimulation in melanoma accessory cells promotes stromal reactivity by inducing pro-inflammatory cytokine production and vasculogenesis, sustaining melanoma growth and aggressiveness, through the ability of pro-inflammatory cytokines to recruit circulating stromal cell precursors (98).

### Hypothesis

#### Is β3-Adrenergic Receptor Functional for Cancer and Fetus Immune Tolerance?

β3-ARs located in the endothelium of human coronary arteries, for example, are 2- to 3-fold more expressed in failing compared with non-failing canine (99) and human hearts (100) and induce an adrenergic-induced vasodilatation through the NO pathway (101). These data suggest that β3-AR upregulation may represent a compensatory mechanism, induced by hypoxia, able to preserve myocardial perfusion during ischemia (101). Similarly, β3-ARs are upregulated in different hypoxic β1 scenarios, such as the mouse model of oxygen-induced retinopathy, the most widely used animal model of retinopathy of prematurity, during the hypoxic phase (102, 103). Also, in this case the demonstration that β3-ARs modulate VEGF release in response to hypoxia through the NO pathway confirms the compensatory mechanism of these receptors, useful to correct retinal hypoxia (104). In conclusion, hypoxia appears to be the ideal environment to induce β3-AR expression, and this is a further similarity between embryo and cancer, where β3-ARs are significantly upregulated under hypoxia conditions (91–98, 105).

Since the involvement of β-ARs in both embryo and cancer development, the similarities between fetal and cancer immune tolerance and, finally, the role, recently demonstrated, of β3-ARs in the promotion of cancer immune escape, we supposed that β3-ARs played a pivotal role also in the regulation of fetal tolerance.

Our recent study, performed in a mouse model of melanoma, has investigated the potential role of β3-ARs in immune-tolerance regulation, evaluating the effect of β-AR blockade on the number and activity of immune cell subpopulations (Treg, NK, CD8, MDSC, macrophages, and neutrophils). First, we described that both β2- and β3-ARs were expressed in mouse peripheral blood mononuclear cells, but only β3-ARs showed a reversible upregulation under hypoxic conditions, followed by a fast downregulation after oxygen re-exposure. Interestingly, β3-ARs were significantly upregulated in NK, Treg, and MDSC infiltrating the tumor if compared with circulating cells. In this study, antagonism of both genetic and pharmacologic β3-ARs reduced melanoma growth *in vivo*, and this effect was concomitant with a significant increase in NK and CD8 number and cytotoxicity and a strong reduction in Treg and MDSC within the tumor mass (105). Treatment with β3-AR antagonists modified the environment rich in M2 macrophages and N2 neutrophils, enhancers of immune escape in an immune-competent M1 and M2 TME. This study did not evaluate specifically the cause–effect relationships between tumor cell death and immune modulation. However, the observation that pretreatment of PBMC under hypoxia with a selective β3-AR antagonist induced an increase in tumor cell death suggests a direct effect of β3-AR present in the immune cell subpopulation (105).

We hypothesize that hypoxia, through the upregulation of β3-AR, promotes the biological shift toward a tolerant immunophenotype and that this is the same trick that embryo and cancer use to create an aura of immune tolerance in a competent immune environment.

## Materials and Methods

### *In vivo* Experiment on Pregnant Mice

*In vivo* experiments were carried out according to the European Union (EU) guidelines for animal care procedures and the Italian legislation (DLgs 26/2014) application of the EU Directive 2010/63/EU. The pregnancy model was established using C57BL/6 mice, co-caging fertile male with adult females overnight. The following morning after the vaginal plug, detection was designated as day 0.5 of pregnancy. Pregnant mice were subcutaneously treated twice a day with SR59230A, CAS: 174689-39-5 (10 mg/kg, Sigma-Aldrich, Saint Louis, MO, United States), or with a physiological solution (vehicle) starting from day 12.5 to day 17.5 of pregnancy. At day 17.5 of pregnancy, 8 dams were sacrificed and the placentas and the maternal deciduae were collected. Briefly, the implantation sites were dissected from the uterus; each placenta/decidua was separated from both the uterine wall and the chorioallantoic membrane and the decidua was gently detached from the placental surface. Eight dams were immediately euthanized after the delivery, and the placentas were rapidly collected and washed with a physiological solution. Placentas were digested in an RPMI 1640 medium containing collagenase D and DNase I for 30 min at 37°C. The total suspension was filtrated through a 70-μm-mesh strainer and centrifuged in conical polypropylene tubes containing Ficoll–Hystopaque. The gradient of mononuclear cells was washed and used for cytofluorimetric analysis.

### Real-Time and Hypoxic Stimulation

For the evaluation of β3-AR expression under normoxic and hypoxic conditions, PBMC were isolated from mouse placental blood with Ficoll–Hystopaque gradient. Then, cells were incubated for 24 h under standard conditions (at 37°C in a humidified incubator with 5% CO2) at 21% O_2_ for normoxia or 1% O_2_ hypoxia. After 24 h, cells were lysed and cDNA was obtained from 500 ng of total RNA using iScript gDNA Clear cDNA Synthesis Kit (Bio-Rad, United States). The expression levels of the *Adrb3* gene were analyzed through quantitative real-time PCR (qRT-PCR) with the use of SsoAdvanced Universal SYBR Green Supermix (Bio-Rad, United States) according to the manufacturer’s instruction and the specific primers (Bio-Rad Assay ID: qMmuCED0001037) in a CFX96 Touch System instrument (Bio-Rad, United States). The normalization was performed using *Tbp* and *Hprt* as housekeeping genes (Bio-Rad Assay ID: qMmuCID0040542 and qMmuCID0005679), and the analysis was done using the ΔΔCt method.

### Flow Cytometry

For the evaluation of β3-AR expression on MDSC, NK, and Treg, cells were isolated from mouse placenta and 50 μl of resuspended cells was marked with β3-AR antibody Ab94506. After 15 min of incubation, cells were washed and resuspended in PBS buffer and marked with 1 μl of FITC-conjugated secondary antibody. Then, cells were washed and resuspended in 200 μl of PBS for FACS analysis.

For MDSC, NK, and Treg marker expression, cells isolated from mouse placenta were incubated and stained with appropriate dilutions of various combinations of the following fluorochrome-conjugated antibodies: anti-CD45-VioBlue or VioGreen (130-110-664, 130-110-665), anti-NKp46-FITC (130-102-300), anti-CD8a-VioBlue (130-102-431), anti-CD3e (17A2)-PE Vio 770 (130-109-839), anti-CD107a-PE (130-102-219), anti-CD161 (NK1.1)-PercCP Vio700 (130-103-963), anti-CD25-PE (130-102-593), anti-CD4-PerCP Vio700 (130-123-213), anti-CD127-APC (130-102-529), anti-CD11b-APC Vio770 (130-109-288), anti-Gr1-PE (130-102-426), anti-CD106-PE (130-116-323), and anti-CD49b-PE (130-108-174). All antibodies were obtained from Miltenyi Biotec, Gladbach, Germany.

Gating strategies for cell detection are reported in Supplementary Figure S1.

### Cell Viability

To distinguish dead from living cells, Viobility 405/520 (120-028-574), and 488/520 (120-028-575) Fixable Dyes obtained from Miltenyi Biotec and analyzed by flow cytometry were used.

### Statistical Analysis

Statistical analysis was performed using GraphPad Prism software by one-way and two-way analysis of variance (ANOVA), followed by the *post hoc* Bonferroni’s test for comparisons of multiple groups. Values are presented as mean ± SEM, *n* = 4 per group. ^∗^*P* < 0.05, ^∗∗^*P* < 0.01, ^∗∗∗^*P* < 0.001, ^****^P < 0.0001, and SR59230a-treated mice compared with vehicles.

## Results

**FIGURE 1:**
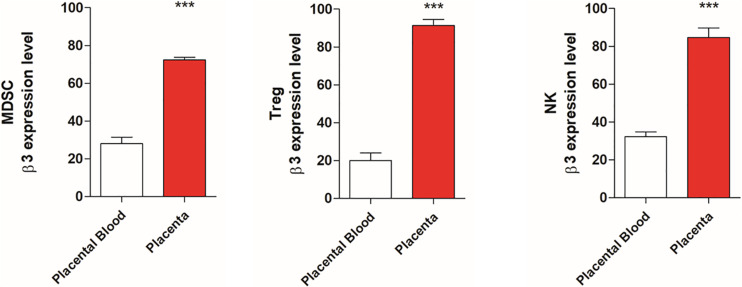
Placental tissues show β3-AR expression increased respect to placental blood samples. FACS quantification of β3-AR expression in NK, Treg, and MDSC. NK (NKp46+/NK1.1 + gated on CD3-/CD45+/β3-AR+), Treg (CD25+/CD127–/β3-AR + gated on CD45+/CD4+) and MDSC (CD11b+, GR1 + β3-AR + gated on CD45+) of placental tissues compared with blood samples of healthy mice. *P < 0.05, **P < 0.01, ***P < 0.001, and ****P < 0.0001, placental tissues compared with placenta blood samples.

Since β3-AR and catecholamine are involved in immune tolerance, we evaluated the expression of this receptor in placental tissues compared with blood samples of healthy mice. Data shown in Figure 1 reveals an increased expression of β3-AR in MDSC, NK, and Treg populations of placenta tissues compared to blood samples.

**FIGURE 2:**
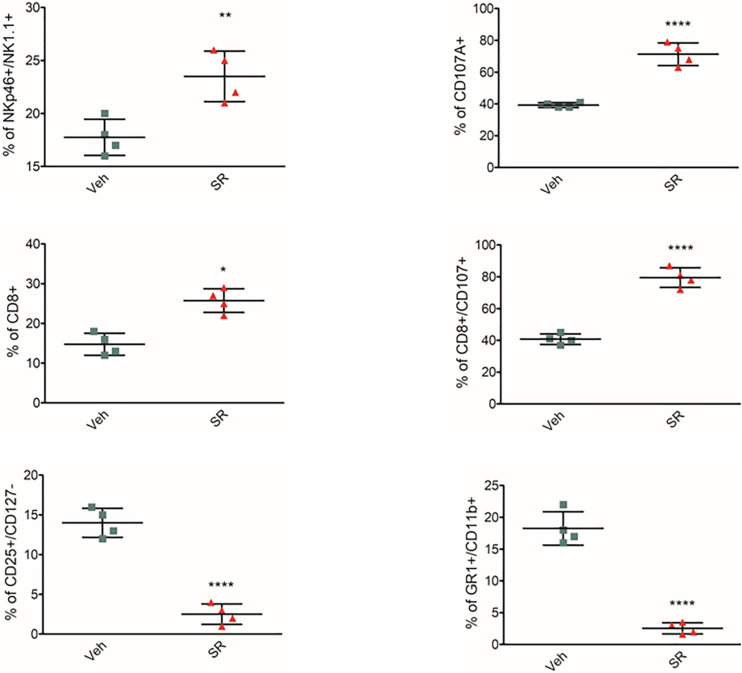
β3-AR antagonism *in vivo* reverts immune-tolerant phenotype in placenta. Analysis of immunologic phenotype in pregnant mouse placenta (*n* = 4) at day 17 of pregnancy. FACS quantification of NK (NKp46+/NK1.1 + gated on CD3-/CD45+), Treg (CD25+/CD127– gated on CD45+/CD4+), and MDSC (CD11b+, GR1 + gated on CD45+) in pregnant mice treated with SR59230a. *P < 0.05, **P < 0.01, and ****P < 0.0001, treated mice compared with vehicle mice.

To identify whether β3-ARs regulate immune tolerance also *in vivo*, female mice at the second week of pregnancy received β3-AR-antagonist SR59230a. Treatment was started on day 12 and continued for 5 days. The animals were sacrificed on day 17. β3-AR blockade increased NK number and cytotoxicity (evaluated by expression of CD107a) and attenuated MDSC and Treg number in mouse placentas (Figure 2). *In vivo* data confirm that β3-ARs support host immune tolerance in the maternal microenvironment by modulating different immune cell populations.

**FIGURE 3:**
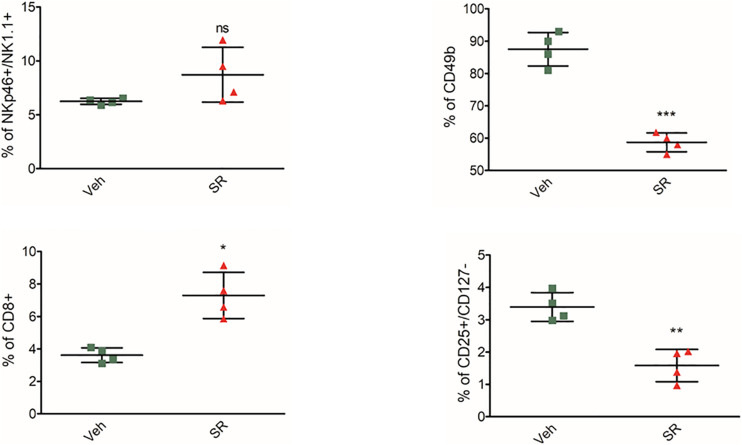
β3-AR antagonism *in vivo* reverts immune-tolerant phenotype in decidua. Analysis of immunologic phenotype in pregnant mouse decidua (*n* = 4) at 2 weeks of pregnancy. FACS quantification of NK (NKp46+/NK1.1 + gated on CD3-/CD45+, CD49b), Treg (CD25+/CD127– gated on CD45+/CD4+) in pregnant mice treated with SR59230a. *P < 0.05, **P < 0.01, and ***P < 0.001, treated mice compared with vehicle mice.

*In vivo* β3-AR blockade had a different effect on decidual cells (Figure 3). There is no significant variation in NK expression. Instead, the deciduous NK and dNK (evaluated by the expression of CD49b) show an opposite trend: their expression is increased with the β3-AR blockade. This response agrees with the different phenotype of the dNK reported in literature. It was not possible to evaluate any changes in decidual MDSC expression because this population was not found. As regards the other populations, the data showed an increase in CD8 and a decrease in Treg cells. The increase in CD8 shows an involvement of T cell toxicity.

**FIGURE 4:**
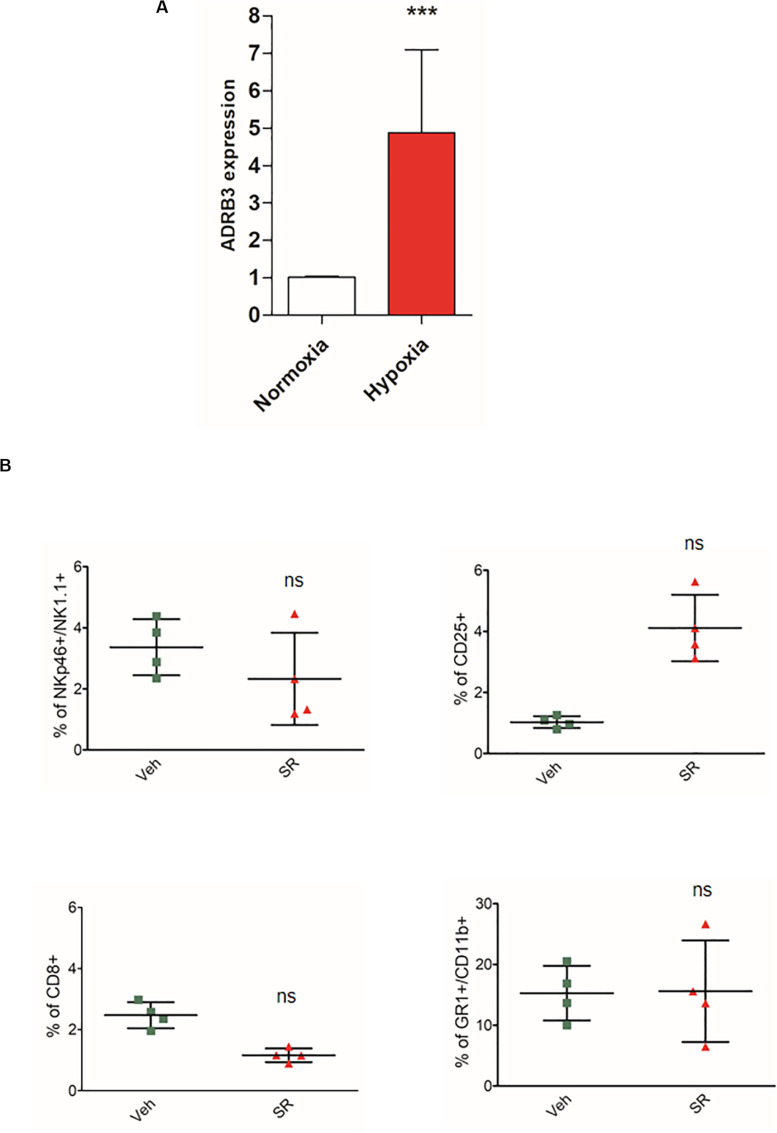
Hypoxia induces an increased expression of *ADRB3* and β3-AR antagonism *in vivo* promotes a different immune-tolerant phenotype in placenta after birth. **(A)** *ABRB3 gene* expression analyzed by quantitative real-time PCR in mice PBMC isolated from placental blood and incubated for 24 h under normoxic (21% O_2_) and hypoxic (1% O_2_) conditions. **(B)** Analysis of immunologic phenotype in pregnant mouse placenta (*n* = 4) after birth. FACS quantification of NK (NKp46+/NK1.1 + gated on CD3-/CD45+), Treg (CD25 + gated on CD45+/CD4+), and MDSC (CD11b+, GR1 + gated on CD45+) in pregnant mice treated with SR59230a. ***P < 0.001, treated mice compared with vehicle mice.

To clarify a possible role on the effect of hypoxia on β3-ARs, we evaluated the expression of the *ADRB3 gene* in mice PBMC through PCR real time. Data reported in Figure 4A show an increase in *ADRB3*.

To demonstrate the crucial role of hypoxia, we decided to repeat the experiment on mouse placentas immediately after birth, to indirectly demonstrate the hypoxic role. Indeed, after birth the effects of hypoxia in the last stages of pregnancy are no longer found. As results showed, SR59230A did not change the immune population compared with the analysis made in placenta at 17 day (Figure 4B).

**FIGURE 5:**
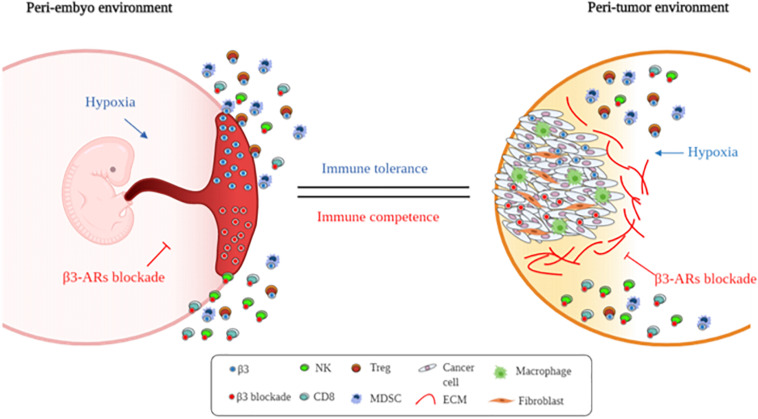
Supposed β3-AR blockade involvement in immune tolerance process. Schematic representation of embryo and cancer immune-tolerance regulation by β3-ARs.

In conclusion, this explorative study suggests that this receptor, usually expressed in hypoxic environments, participates in the local origin of fetal immune tolerance (Figure 5). Further studies need to be conducted for understanding the real role of β3-ARs.

## Discussion

Recently, β3-ARs have been demonstrated to be involved in cancer-related immune tolerance under hypoxic conditions (105). It is well known that hypoxia plays a crucial role in fetus development and in cancer progression, participating in processes such as angiogenesis, apoptosis, cell migration, invasion, and metastasis (106, 107). Actually, early human placental tissue develops in a physiologically hypoxic environment, such as required to induce specific placental metabolic activities (108). Moreover, β3-ARs are upregulated and represent the predominant subtype over β2-ARs in the human pregnant myometrium (87), where they inhibit spontaneous contractions (88). Here we postulate that the relationship linking hypoxic upregulation of β3-ARs and promotion of immune tolerance recently demonstrated to enhance cancer progression (105) actually follows the same mechanisms originally foreseen to guarantee fetal tolerance.

Since β3-ARs is involved in various hypoxic scenarios in pathological and physiological states, including pregnancy, in this work we have shown that β3-ARs is strongly induced in the immune subpopulations responsible for immune tolerance and that occurs because the intrauterine environment is hypoxic.

In this respect, we have recently demonstrated that β3-ARs are actively involved in all the different scenarios where hypoxia induces important steps necessary to ensure progression of cancer and/or embryo.

β3-ARs, in fact, participate in the promotion of angiogenesis (necessary for tumor progression but also for placenta development), through an axis NO-VEGF mediated (95–98, 105).

Recently, our studies showed that β3-ARs are actively involved in the stimulation of a metabolic shift (necessary in the development of a metabolism specifically programmed to live in a hypoxic environment) through the promotion of aerobic glycolysis (109), yet another common feature shared by early preimplantation mammalian embryo (110), decidua during early pregnancy (111), and tumors (112, 113). Both cancer cells and embryos increase the uptake of glucose and the expression of glycolytic enzymes to obtain energy for growth (Warburg effect). This metabolic shift favors their proliferative activity since this metabolic pathway produces a large number of useful intermediates to secondary biosynthetic pathways and induces an increased export of lactate, useful to facilitate the trophoblast or tumor infiltration (114). It has been reported that both in cancer and embryonic stem cells, β3-ARs promote this metabolic shift, not only inducing the specific glycolytic cytoplasmic enzymes but also promoting the expression of UCP-2 (uncoupling protein-2) responsible for a reduced mitochondrial activity and inhibition of mitochondrial reactive oxygen species production (109). Interestingly, β3-ARs are highly expressed in cancer stem cells, and our studies in melanoma have clearly demonstrated that β3-ARs are involved in the enhancement stem-like traits, such as CD133, and CD20 expression and P1 melanosphere formation (98).

More recently, β3-ARs have been demonstrated to be related with the maintenance of an undifferentiated state also in neuroblastoma cells (115). These data are in line with the demonstration that β3-ARs are precociously expressed in the first phases of embryogenesis (84). We hypothesize that during the first phases of embryogenesis, the strong hypoxia induces a precocious expression of β3-ARs that maintains embryo in an undifferentiated state. As pregnancy evolves, the placentation induces a progressive increase in oxygen levels, and this represents the signal for a reduction in the expression of β3-ARs, and therefore, the induction of differentiation. Therefore, β3-ARs appear again to play a similar role both in cancer and embryo. Finally, this study provides the first data demonstrating how β3-AR blockage can modulate distinct immune cell populations involved in the immune tolerance process during pregnancy. These data are consistent with those recently demonstrated around and within the tumor (105). If these data will be confirmed and supported by further experiments (for example in the early stages of pregnancy), it will be possible to imagine a decisive role of β3-AR in promoting fetal and tumor immune tolerance.

### Limitations and Perspectives

There are several limitations in this study.

The exploratory nature of this research, aimed at evaluating a possible role of the β3-ARs in the modulation of the cells involved in fetal immunotolerance, is confirmed by the limited number of animals involved. It is therefore evident that a much larger number of experiments are required to confirm the reproducibility of our data.

Inbred mice were chosen because of the high reproducibility of results that allowed reducing sample size, and therefore the number of animals used (116). The choice of this animal model deserves criticism. In fact, this study was performed on a simplified pregnancy model, between syngeneic animals with restricted polygenic diversity. However, in C57BL/6J pregnant mice, immune tolerance is preserved and therefore this mouse strain can represent a valid model for exploratory studies (117). Rather, the demonstration that the blockade of β3-ARs induces a sensitive modulation of the cells involved in fetal immune tolerance in this “low immunologic impact” model could suggest an even more relevant impact in allogeneic pregnancies. Also in this case, the exploratory role of this study is evident, and it therefore becomes necessary to repeat this study in allogeneic pregnancies.

In this study, we decided to treat mice with the β3-AR antagonist during the second week of gestation. Also, this choice may appear legitimately questionable and criticizable, especially if our hypothesis envisages hypoxia as a trigger for modulating the immune phenotype. In fact, if the oxygenation of the murine placenta behaved like the human placenta, with a positive correlation between placental oxygenation and gestational age, our hypothesis should be tested at an early stage of pregnancy (108). However, the oxygenation of the murine placenta does not undergo particular variations in the period between 10 and 18 days of pregnancy (118). In contrast, the lowest oxygen values appear to be observed around the eighteenth day of pregnancy (119). These observations therefore legitimize our choice of intervention timing.

Finally, the adoption of this model did not make it possible to evaluate whether β3-AR blockade at an early stage of pregnancy could induce an increased abortion rate, essential information to evaluate the relevance of this receptor for tolerance induction *in vivo* and to evaluate a possible role in the implantation phase.

The significant limitations of this study require further investigation with a larger number of experiments.

## Conclusion

In conclusion, this study presents a new hypothesis and a new interpretation on the development of fetal and tumor immune tolerance. Cancer appears to promote immune tolerance by using the same molecular strategy (mainly β3-AR-mediated) adopted by the embryo and fetus. In this light, TME might act like placental tissue, and cancer might be a disease that exploits the same strategies that allow the embryo to grow. Furthermore, this study indicates that the TME reactivates fetal competences, including immunosuppression, predominantly through the activation of β3-ARs.

Although clinical benefits are currently expected by the addition of available non-selective β-blockers, in the near future β3-AR blockade could represent a more effective strategy to overcome immunoediting.

## Data Availability Statement

The datasets generated for this study are available on request to the corresponding author.

## Ethics Statement

The animal study was reviewed and approved by Research permit #194/2015-PR approved by the Italian Ministry of Health.

## Author Contributions

LF and MC developed the concept and experiments and wrote the manuscript. AD, AS, DB, VD, SC, AP, and PN performed and analyzed animal model and functional assays. CF revised the experiments and the manuscript.

## Conflict of Interest

The authors declare that the research was conducted in the absence of any commercial or financial relationships that could be construed as a potential conflict of interest.

## Supplementary Material

FIGURE S1 Gating strategy of FACS analysis. Representative gating strategy on whole blood, placenta and decidua for NK, MDSC, Treg, and CD8 analysis.

## Abbreviations

β-ARs: β-adrenergic receptors
APC: antigen-presenting cells
cNK: conventional natural killer
COX-2: cyclooxygenase-2
CTLA-4: cytotoxic T-lymphocyte antigen-4
dNK: decidual NK
FasL: Fas ligand
HLAs: human leukocyte antigens
IDO: indoleamine-2,3-dioxygenase
MDSC: myeloid derived suppressor cells
NK: natural killer
NKG2DL: NKG2D ligand
NFkB: nuclear factor-k B
PD-L1: programmed cell death-1 (PD-1)/PD-ligand 1
Treg: regulatory T cells
TGF-β: transforming growth factor-β
TME: tumor microenvironment.
