# Supplementary figures and images for: β3-Adrenoceptors as Putative Regulator of Immune Tolerance in Cancer and Pregnancy
Source: Front Immunol. 2020 Sep 2;11:2098. doi: 10.3389/fimmu.2020.02098 (PMC7492666; doi:10.3389/fimmu.2020.02098)

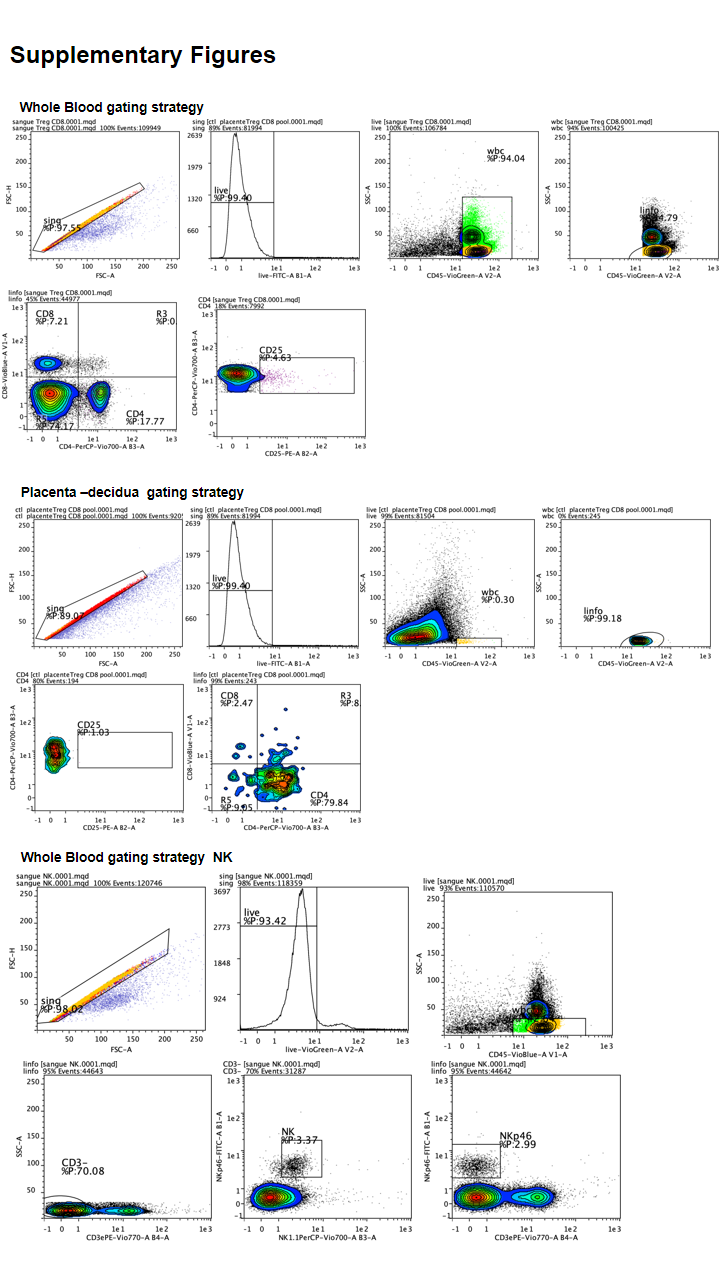

Supplement: FIGURE S1 — Gating strategy of FACS analysis. Representative gating strategy on whole blood, placenta and decidua for NK, MDSC, Treg, and CD8 analysis. [file Image_1.TIF]

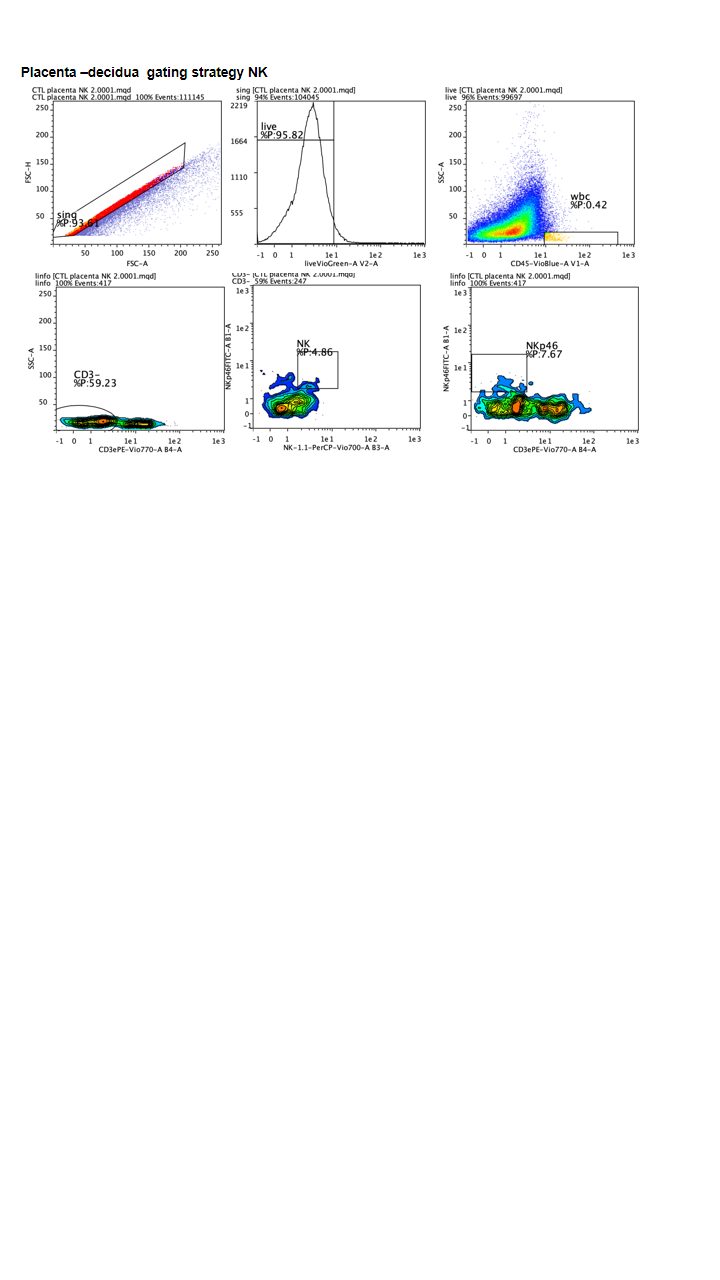

Supplement: Supplementary file 2 [file Image_2.TIF]
